# Prostaglandin E_2_ Dilates Intracerebral Arterioles When Applied to Capillaries: Implications for Small Vessel Diseases

**DOI:** 10.3389/fnagi.2021.695965

**Published:** 2021-08-13

**Authors:** Amanda C. Rosehart, Thomas A. Longden, Nick Weir, Jackson T. Fontaine, Anne Joutel, Fabrice Dabertrand

**Affiliations:** ^1^Department of Anesthesiology, University of Colorado Anschutz Medical Campus, Aurora, CO, United States; ^2^Department of Physiology, School of Medicine, University of Maryland, Baltimore, Baltimore, MD, United States; ^3^INSERM, UMR 1266, GHU Paris Psychiatrie et Neurosciences, Institute of Psychiatry and Neuroscience of Paris, University of Paris, Paris, France; ^4^Department of Pharmacology, Larner College of Medicine, University of Vermont, Burlington, VT, United States; ^5^Department of Pharmacology, University of Colorado Anschutz Medical Campus, Aurora, CO, United States

**Keywords:** functional hyperemia, cerebral small vessel diseases, CADASIL, microcirculation, neurovascular coupling, potassium channel, prostaglandin E_2_, epidermal growth factor receptor

## Abstract

Prostaglandin E_2_ (PGE_2_) has been widely proposed to mediate neurovascular coupling by dilating brain parenchymal arterioles through activation of prostanoid EP4 receptors. However, our previous report that direct application of PGE_2_ induces an EP1-mediated constriction strongly argues against its direct action on arterioles during neurovascular coupling, the mechanisms sustaining functional hyperemia. Recent advances have highlighted the role of capillaries in sensing neuronal activity and propagating vasodilatory signals to the upstream penetrating parenchymal arteriole. Here, we examined the effect of capillary stimulation with PGE_2_ on upstream arteriolar diameter using an *ex vivo* capillary-parenchymal arteriole preparation and *in vivo* cerebral blood flow measurements with two-photon laser-scanning microscopy. We found that PGE_2_ caused upstream arteriolar dilation when applied onto capillaries with an EC_50_ of 70 nM. The response was inhibited by EP1 receptor antagonist and was greatly reduced, but not abolished, by blocking the strong inward-rectifier K^+^ channel. We further observed a blunted dilatory response to capillary stimulation with PGE_2_ in a genetic mouse model of cerebral small vessel disease with impaired functional hyperemia. This evidence casts previous findings in a different light, indicating that capillaries are the locus of PGE_2_ action to induce upstream arteriolar dilation in the control of brain blood flow, thereby providing a paradigm-shifting view that nonetheless remains coherent with the broad contours of a substantial body of existing literature.

## Introduction

As a leading cause of stroke and dementia, cerebral small vessel diseases (SVDs) pose a horrendous threat to the elderly population (Pantoni, 2010; Wardlaw et al., 2019). Despite their major contribution to age-related vascular cognitive impairment and disability (Iadecola et al., 2019), the disease processes and key biological mechanisms underlying these disorders remain largely unknown. Moreover, there are no specific treatments outside the management of vascular risk factors and use of anti-platelets after ischemic stroke. However, accumulating experimental evidence suggests that functional alterations in the cerebral microcirculation have early and deleterious consequences on functional hyperemia—the ability of the brain to increase local blood flow in response to local increases in neuronal activity (Huneau et al., 2018; Iadecola et al., 2019).

Cerebral Autosomal Dominant Arteriopathy with Subcortical Infarcts and Leukoencephalopathy (CADASIL) is the most common genetic form of cerebral SVDs. Remarkably, CADASIL includes all clinical and MRI manifestations of sporadic SVDs, hence offering a lens through which to view more common forms of sporadic SVDs (Chabriat et al., 2009). Both CADASIL patients and the *TgNotch3^*R169C*^* mouse model, hereafter referred to as SVD mice, exhibit deficits in functional hyperemia at an early stage of the disease progression (Joutel et al., 2010; Huneau et al., 2018). We have recently demonstrated that activation of the epidermal growth factor receptor (EGFR) by its ligand heparin-binding EGF-like growth factor (HB-EGF) ameliorates the cerebral vascular deficits of the SVD mouse—including impaired functional hyperemia (Capone et al., 2016; Dabertrand et al., 2021). Part of this effect is mediated by reenabling capillary-to-arteriole signaling during the neurovascular coupling that underpins functional hyperemia (Dabertrand et al., 2021). In physiological conditions, action potentials increase extracellular K^+^ concentration which activates the strong inward-rectifier K^+^ (Kir2.1) channel in capillary endothelial cells (cECs). This creates a hyperpolarizing signal that rapidly propagates to upstream arterioles, driving vasodilation and local hyperemia (Longden et al., 2017; Harraz et al., 2018a; Moshkforoush et al., 2020). This mechanism is vulnerable to pathology, and we recently demonstrated that SVD lowers the synthesis of the phospholipid PIP_2_, which prevents Kir2.1 channels from acting as sensors of increases in external K^+^ (Dabertrand et al., 2021). Strikingly, we showed that both HB-EGF and systemic injection of exogenous PIP_2_ improved functional hyperemia deficits in SVD mice by restoring capillary-to-arteriole signaling.

Functional hyperemia is a vital process controlled by multiple mechanisms that provide layers of redundancy, and thus neurovascular coupling agents other than K^+^ ions have been postulated (Attwell et al., 2010; Kaplan et al., 2020). Among these, prostaglandin E_2_ (PGE_2_), produced by cyclooxygenase-2 (COX-2) from arachidonic acid, has been widely proposed to be released from excitatory neurons to relax arteriolar smooth muscle cells (SMCs) by activation of the G_*S*_ protein-coupled prostanoid EP4 receptor and subsequent cyclic adenosine monophosphate-dependent pathway (Zonta et al., 2002; Takano et al., 2005; Gordon et al., 2008; Attwell et al., 2010; Lacroix et al., 2015). Yet, this interpretation appears incompatible with our previous demonstration that PGE_2_ causes constriction, rather than dilation, when applied directly to isolated cortical arterioles—an effect that occurs via activation of the G_*q*_ protein-coupled prostanoid EP1 receptor (Dabertrand et al., 2013). Consistent with these observations, the EP1 receptor is robustly expressed not only in the arteriolar SMCs, but also in cECs of the brain microcirculation (Vanlandewijck et al., 2018). We therefore hypothesized that PGE_2_ contributes to neurovascular coupling by initiating a vasodilatory signal from the capillaries to the upstream parenchymal arterioles, rather than by targeting SMCs directly. Using a combination of *ex vivo* and *in vivo* approaches, we report that capillary stimulation with PGE_2_ induces upstream arteriolar dilation and increases blood flow *in vivo*. Consistent with defective functional hyperemia, we further show that PGE_2_-induced capillary-to-arteriole signaling is blunted in the SVD mouse model and can be rescued by HB-EGF.

## Materials and Methods

### Animals

All experimental protocols used in this study were approved by the Institutional Animal Care and Use Committee (IACUC) of the University of Colorado, Anschutz Medical Campus. Adult (2–3 months old) male C57/BL6J mice (Jackson Laboratories, United States), were housed on a 12-h light:dark cycle with environmental enrichment and free access to food and water. *TgNotch3*^*WT*^ (WT) and *TgNotch3^*R169C*^* (SVD) lines have been previously described (Joutel et al., 2010) and were used at 6 months of age in order for the *TgNotch3^*R169C*^* mice to develop the SVD phenotype, and for consistency with our previous studies (Dabertrand et al., 2015, 2021; Capone et al., 2016; Fontaine et al., 2020). All mice were euthanized by i.p. injection of sodium pentobarbital (100 mg/kg) followed by rapid decapitation.

### *Ex vivo* Capillary-Parenchymal Arteriole Preparation

The CaPA preparation was obtained as previously described (Longden et al., 2017; Rosehart et al., 2019) by dissecting intracerebral arterioles arising from the M1 region of the middle cerebral artery, leaving the attached capillary bed intact. Arteriolar segments were cannulated on glass micropipettes with one end occluded by a tie and pressurized using a Living Systems Instrumentation (United States) pressure servo controller with a mini peristaltic pump. The ends of the capillaries were then sealed by the downward pressure of an overlying glass micropipette. CaPA preparations were superfused (4 mL/min) with prewarmed (36°C ± 1°C), gassed (5% CO_2_, 20% O_2_, and 75% N_2_) artificial cerebrospinal fluid (aCSF) for at least 30 min. The composition of aCSF was 125 mM NaCl, 3 mM KCl, 26 mM NaHCO_3_, 1.25 mM NaH_2_PO_4_, 1 mM MgCl_2_, 4 mM glucose, 2 mM CaCl_2_, pH 7.3 (after aeration with 5% CO_2_). Application of pressure (40 mmHg) to the cannulated parenchymal arteriole segment in this preparation pressurized the entire tree and induced myogenic tone in the arteriolar segment. Only viable CaPA preparations, defined as those that developed pressure-induced myogenic tone greater than 15%, were used in subsequent experiments. Arteriolar viability was validated by bath-applying NS309 (1 μM), which causes an endothelial-dependent vasodilation through activation of small- and intermediate-conductance, Ca^2+^-sensitive K^+^ (SK and IK, respectively) channels, or the thromboxane receptor agonist U46619 (1 μM), which causes robust vasoconstriction. Dilatory responses of the attached arteriole segment to K^+^ and PGE_2_ were obtained by applying aCSF containing 10 mM K^+^ or PGE_2_ onto capillary extremities by pressure ejection from a glass micropipette (tip diameter, ∼5 μm) attached to a Picospritzer III (Parker, United States) at ∼5 psi for 20 s. The dose-responses were performed using pipettes filled with the different concentrations of PGE_2_ and testing them sequentially starting with the lowest concentration. Contrary to arteriolar endothelial cells, cECs do not express functional IK and SK channels and spatial restriction of the drugs applied onto the capillary ends was validated by the lack of response to local stimulation with NS309 (1 μM), as previously described (Rosehart et al., 2019). In some control experiments, K^+^ and PGE_2_ were applied directly to the arteriole segment and the other drugs were applied via the bath perfusion. The luminal diameter of the parenchymal arteriole was acquired at 15 Hz using a CCD camera and IonWizard 6.2 edge-detection software (IonOptix, United States). Two regions were simultaneously recorded, zone 1 where the capillary tree sprouted from of the arteriole and zone 2 located 250 μm upstream of this, and diameter from both of these sites was averaged unless noted otherwise. Changes in arteriolar diameter were calculated from the average luminal diameter measured over the last 10 s of stimulation and were normalized to the maximum dilatory responses in 0 mM Ca^2+^ bath solution at the end of each experiment using the following equation: [(change in diameter)/(maximal diameter-initial diameter)] × 100.

### *In vivo* Imaging of Cerebral Hemodynamics

**FIGURE 1:**
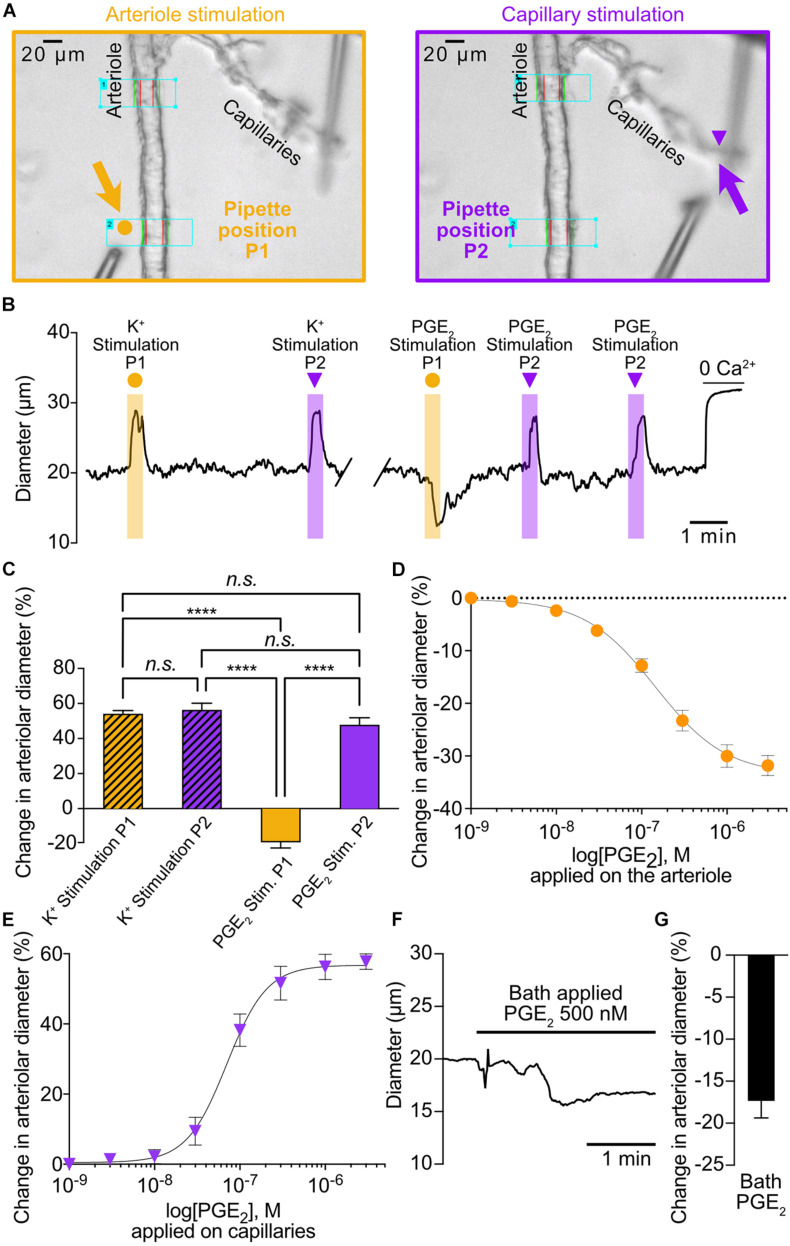
PGE_2_ causes upstream arteriolar dilation when applied onto capillaries. **(A)** Pipette positions for arteriole stimulation (left, 5 orange arrow) and capillary stimulation (right, purple arrow) in CaPA preparations. **(B)** Representative trace of arteriolar diameter showing effects of pressure ejection of 10 mM K^+^ or 1 μM PGE_2_ onto arteriole (P1, orange dot) and capillaries (P2, purple triangle). **(C)** Summary data from 6 mice (*n.s.*, not significant; ****P < 0.0001, one-way ANOVA, Tukey’s test). **(D)** Concentration-response curve produced by locally applying PGE_2_ to the arteriolar segment over a concentration range of 1 nM to 3 μM (5 mice). An EC_50_ of 145 nM was calculated from the non-linear regression curve. **(E)** Concentration-response curve produced by locally applying PGE_2_ to capillary extremities over a concentration range of 1 nM to 3 μM (8 mice). An EC_50_ of 70 nM was calculated from the non-linear regression curve. **(F)** Representative trace of arteriolar diameter showing effects of bath application of 500 nM PGE_2_. **(G)** Summary data from 5 mice.

As previously described (Longden et al., 2017), mice were anesthetized with isoflurane (5% induction and 2% maintenance) during the surgical procedure. The skull was exposed, cleaned, and a stainless-steel head plate was attached with a mixture of dental cement and superglue. Isoflurane was replaced with α-chloralose (50 mg/kg) and urethane (750 mg/kg) during recording. FITC-dextran (2000 kDa) was injected via the retro-orbital sinus to visualize the cerebral vasculature and for contrast imaging of RBCs. A penetrating arteriole, identified by the direction of the RBCs flowing into the brain, was followed and a downstream capillary was selected for study in cortical layers 2 or 3. A pipette was then maneuvered into the brain and positioned adjacent to the capillary under study, and aCSF containing, or not, 1 μM PGE_2_ was ejected (200–300 ms, 8 ± 1 psi, ∼4 picoliters). The ejected volume was monitored by including 0.2 mg/mL tetramethylrhodamine isothiocyanate (TRITC; 150 kDa)-labeled dextran in the pipette (Figure 1E). RBC flux data were collected by line scanning at 5 kHz. Images were acquired using a Zeiss LSM-7 multiphoton microscope (Zeiss, United States) equipped with a 20x Plan Apochromat 1.0 N.A. DIC VIS-IR water-immersion objective and coupled to a Coherent Chameleon Vision II Titanium-Sapphire pulsed infrared laser (Coherent, United States). FITC and TRITC were excited at 820 nm, and emitted fluorescence was separated through 500–550 and 570–610 nm bandpass filters, respectively.

### Reagents

HC030031, HC067047, PGE_2_, NS309, and SC51322 were purchased from Tocris Bioscience (United States); All other chemicals and reagents were obtained from Sigma-Aldrich (United States). The vehicle for HB-EGF solutions was 0.2-μm–filtered PBS containing 0.1% BSA.

### Statistical Analysis

Data in figures and text are presented as means ± standard error of the mean (SEM). Statistical testing was performed using GraphPad Prism 8 software. All data passed the Kolmogorov–Smirnov test for normality. Statistical significance was determined using one-way analysis of variance (ANOVA) followed by Tukey’s *post hoc* test, unless otherwise stated.

## Results

To investigate the effect of capillary stimulation with PGE_2_ on upstream arteriolar diameter, we used our previously developed *ex vivo* capillary-parenchymal arteriole (CaPA) preparation that allows to apply vasoactive substances at specific points along the arteriole-capillary continuum by pressure ejection (Longden et al., 2017; Rosehart et al., 2019). Consistent with our previous report that PGE_2_ acts as a vasoconstrictor (Dabertrand et al., 2013), local application of 1 μM PGE_2_ directly on the arteriolar segment caused a decrease in diameter (Figures 1A–C). In contrast, and as expected (Longden et al., 2017; Dabertrand et al., 2021), 10 mM K^+^ also applied on the arteriole caused a robust dilation. Interestingly, either 10 mM K^+^ or 1 μM PGE_2_ caused upstream dilations when applied onto capillary extremities and the amplitudes of these were virtually identical (56.2% ± 3.9% and 47.8% ± 4.2%, respectively) (Figures 1A–C and Supplementary Movie 1). However, the kinetics of the responses appear significantly different with slower onset (5.27 ± 1.35 s) and time to peak (9.65 ± 1.5 s) for PGE_2_ compared to K^+^ responses, (1.7 ± 0.4 s and 3.87 ± 1.13 s, respectively).

We next tested the effect of increasing concentrations of PGE_2_ locally applied to the arteriolar segment and observed a concentration-dependent constriction with a calculated EC_50_ of 145 nM (Figure 1D). The arteriolar dilation in response to capillary stimulations with PGE_2_ was also concentration-dependent with a calculated EC_50_ of 70 nM and a maximal response at 1 μM, and thus we chose to use the latter concentration throughout the study for capillary stimulation (Figure 1E and Supplementary Movie 1). Finally, we tested the effect of a modest concentration of PGE_2_ (500 nM) applied via the bath perfusion, hence stimulating both the capillary ends and the arteriolar segment, and measured a small but clear constriction (Figures 1F,G).

**FIGURE 2:**
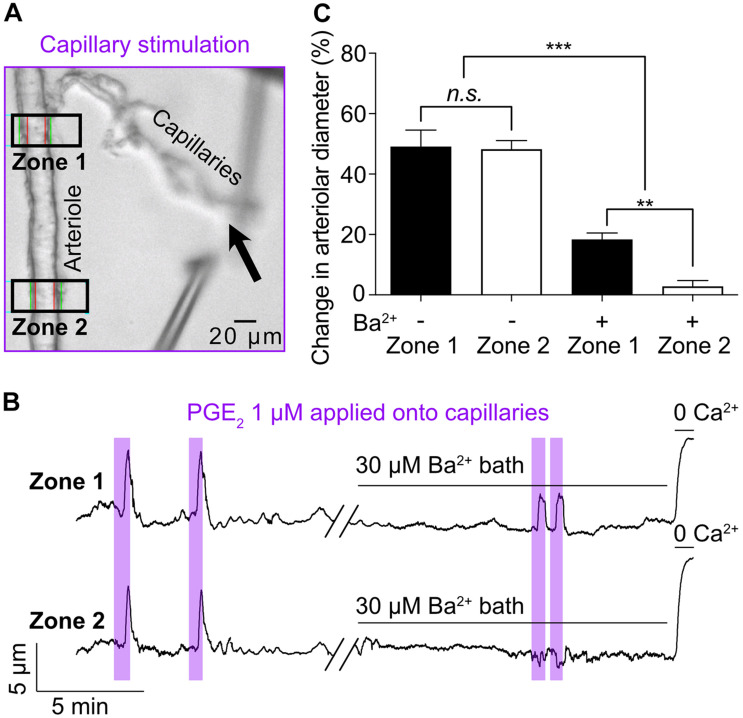
Kir channel blocker Ba^2+^ (30 μM) inhibits the propagation, not the initiation, of the PGE_2_-induced upstream arteriolar dilation. **(A)** Micrograph showing Zone 1 and Zone 2 positions for arteriolar diameter recording during capillaries stimulation (black arrow) with 1 μM PGE_2_. **(B)** Representative traces of arteriolar diameter at zone 1 and zone 2 showing the effect of 30 μM bath-applied Ba^2+^ on PGE_2_-induced upstream dilation. **(C)** Summary data from 5 mice (*n.s.*, not significant; **P < 0.01, ***P < 0.001, one-way ANOVA, Tukey’s test).

In our initial report of capillary-to-arteriole electrical signaling (Longden et al., 2017), we identified the cEC strong inward-rectifier K^+^ channel, Kir2.1, as the molecular cornerstone of the capillary-to-arteriole electrical mechanism elicited by capillary stimulation with 10 mM K^+^. Increasing extracellular K^+^ concentration from 3 to 10 mM activates Kir2.1 channels in cECs, which induces a regenerative hyperpolarization that travels retrogradely to dilate the upstream arteriole. Consistent with this model, 30 μM Ba^2+^—which, among the inward rectifier K^+^ channels expressed in the microcirculation, preferentially blocks Kir2 channels (Longden and Nelson, 2015)—completely abolished the arteriolar dilation in response to capillary-applied 10 mM K^+^. Therefore, we tested the effect of Ba^2+^ on arteriolar dilation induced by capillary stimulation with PGE_2_. Arteriolar diameter was recorded at two distinct zones: zone 1 was placed at the branching of the transitional segment from the arteriole and zone 2 was located 250 μm upstream of this (Figure 2A). PGE_2_ locally applied onto capillaries led to similar dilatory responses in zone 1 (49.1% ± 5.5%) and zone 2 (48.2% ± 2.9%) (Figures 2B,C). Remarkably, bath application of 30 μM Ba^2+^ decreased upstream arteriolar dilation at zone 1 in response to capillary stimulation with PGE_2_ by 63% and virtually eliminated dilation at zone 2. The onset of the remnant dilation observed at zone 1 was unchanged by Ba^2+^ (5.27 ± 1.35 s 6.49 ± 2.81 s). Taken together, these data suggest that the Kir2.1 channel is not required to initiate PGE_2_-induced upstream arteriolar dilation, but rather participates in the amplification and propagation of the vasodilatory signal, as evidenced by the difference between zone 1 and zone 2.

**FIGURE 3:**
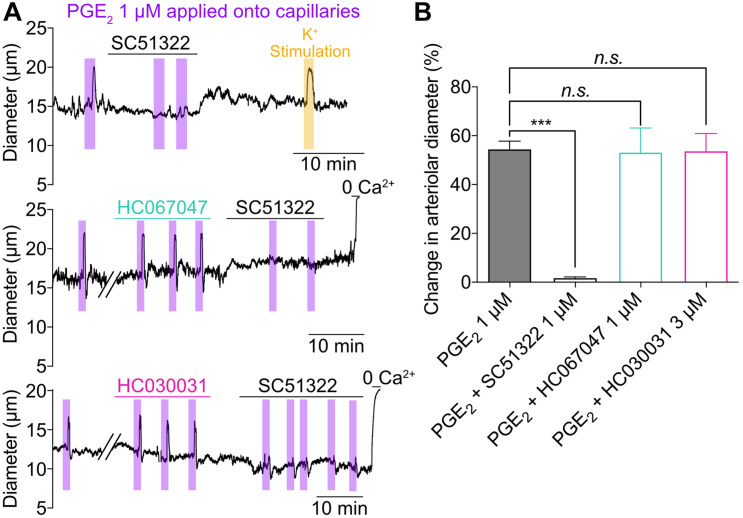
Activation of capillary EP1 receptor initiates PGE_2_-induced upstream dilation independently of TRPV4 and TRPA1 channels. **(A)** Representative traces of arteriolar diameter showing effects of pressure ejection of PGE_2_ 1 μM onto capillaries in absence or presence of SC51322 (1 μM, EP1 antagonist), HC067047 (1 μM, TRPV4 antagonist), and HC030031 (3 μM, TRPA1 antagonist). **(B)** Summary data from 5 to 8 mice (*n.s.*, not significant; ***P < 0.001, one-way ANOVA, Dunnett’s test).

To investigate the mechanistic underpinnings of the capillary-to-arteriole signaling induced by PGE_2_, we first superfused the *ex vivo* CaPA preparation with 1 μM SC51322, a prostanoid receptor antagonist specific for the EP1 receptor. SC51322 abolished the response to PGE_2_, while the dilation induced by 10 mM K^+^ remained intact (Figures 3A,B). This observation suggests that PGE_2_ acts through activation of G protein-coupled receptors of the G_*q/*__11_ subtype (G_*q*_PCR) and subsequent Ca^2+^ signaling. Depletion of plasma membrane phosphatidylinositol 4,5-bisphosphate (PIP_2_) following G_*q*_PCR activation is known to stimulate transient receptor potential (TRP) channels, a major pathway for extracellular Ca^2+^ influx (Kim et al., 2008; Harraz et al., 2018b). Moreover, recent work by Thakore et al. (2021) has revealed that activation of TRPA1 channel in cECs can initiate a biphasic, propagating retrograde signal that dilates upstream parenchymal arterioles during functional hyperemia. However, the TRPA1 antagonist HC030031 at 3 μM had no effect on the dilation induced by capillary stimulation with PGE_2_ (Figures 3A,B). Inhibition of TRPV4, another Ca^2+^-permeable TRP channel expressed by cECs (Harraz et al., 2018b), with 1 μM HC067047 also did not impact the effect of PGE_2_ (Figures 3A,B). These results suggest that PGE_2_ induces upstream arteriole dilation via activation of the EP1 receptor but independently of TRPV4 and TRPA1 channels.

**FIGURE 4:**
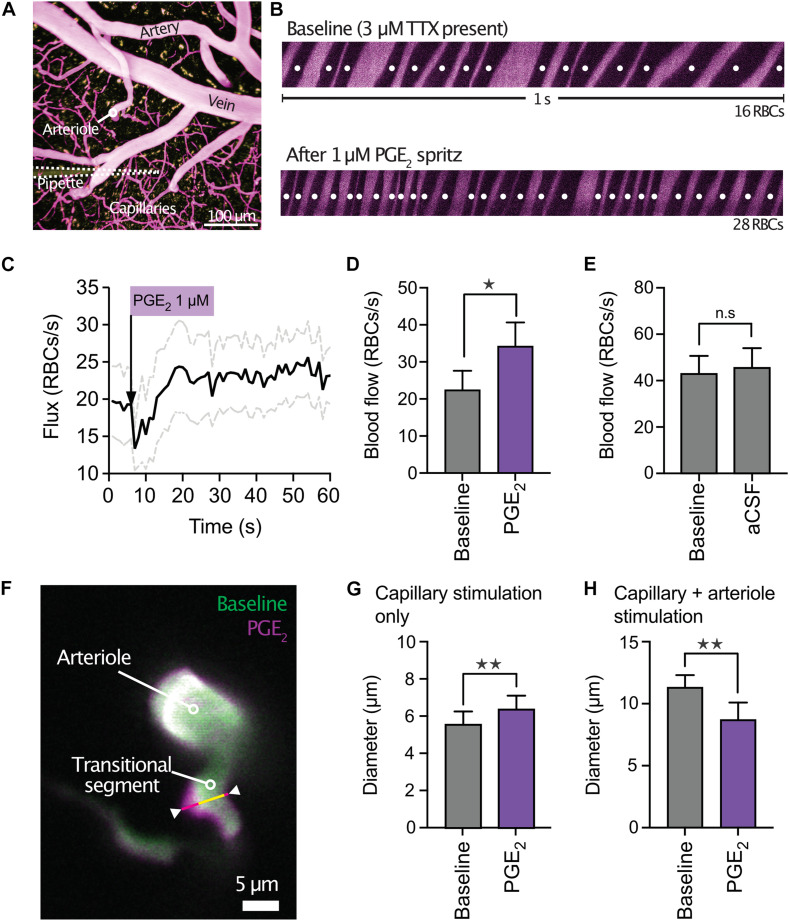
PGE_2_ causes capillary hyperemia *in vivo*. **(A)** Micrograph of mouse cortical vasculature showing a micropipette containing PGE_2_ and TRITC-dextran (yellow) in close apposition to a capillary (FITC-dextran, purple). **(B)** Red blood cell (RBC) flux was measured by high-frequency line scanning over a period of 1 min at baseline (top panel) and after application of 1 μM PGE_2_ (lower panel) onto a capillary. RBCs appear as black streaks in plasma (purple). **(C)** Average traces (black line) plus SEM (gray lines) showing the increase in RBC flux to PGE_2_. The dip immediately following the ejection of PGE_2_ is caused by momentary pressure on the capillary wall. **(D)** Summary data of RBC flux showing significant hyperemia following capillary stimulation with 1 μM PGE_2_ (*n* = 14 experiments, 6 mice, *P < 0.05, paired Student’s *t*-test). **(E)** In contrast, ejection of aCSF vehicle onto capillaries had no effect on RBC flux (*n* = 9 experiments, 5 mice, *P* > 0.05, paired Student’s *t*-test). **(F)** Overlay showing the diameter of a penetrating arteriole and the transitional segment to the capillary bed at baseline (green) and after PGE_2_ ejection onto a downstream capillary (magenta). The upstream dilation was most prominent in the region highlighted by the white arrowheads. **(G)** Summary data showing upstream dilation to capillary ejection of PGE_2_ (*n* = 5 experiments, 4 mice, **P < 0.01, paired Student’s *t*-test). **(H)** In contrast, simultaneous stimulation of both capillaries and arterioles with PGE_2_ *in vivo* led to constriction (*n* = 7 experiments, 3 mice, **P < 0.01, paired Student’s *t*-test).

We then tested the ability of PGE_2_ to dilate upstream arterioles *in vivo* by measuring capillary red blood cell (RBC) flux with two-photon laser-scanning microscopy. Fluorescein isothiocyanate (FITC)-labeled dextran was used to visualize the cortical microcirculation and a pipette containing 1 μM PGE_2_ was maneuvered into the brain through a cranial window and positioned next to a capillary of interest (Figure 4A). Pressure ejection (8 ± 1 psi) for up to 300 ms of PGE_2_ evoked a significant increase in capillary RBC flux (Δ = 12 ± 4 RBCs/s) consistent with the notion that the mechanisms we observed in CaPA preparations are also at play in the intact system (Figures 4B–D). In contrast, ejection of aCSF vehicle had no effect on capillary RBC flux (Figure 4E). To determine whether PGE_2_ also causes upstream dilation we next performed experiments in which we imaged at the parenchymal arteriole-transitional zone junction (Figure 4F). Here, we found that ejection of 1 μM PGE_2_ selectively onto capillaries routinely produced a small upstream dilation (Figures 4F,G and Supplementary Movie 2). In contrast, if we applied PGE_2_ onto both capillaries and arterioles *in vivo* by increasing the duration of ejection, causing spread of the ejected solution to the upstream arteriole along a paravascular route, we observed constriction (Figure 4H), consistent with our *ex vivo* data. As expected, ejection of aCSF vehicle alone had no effect on upstream arteriolar diameter (12.95 ± 0.57 μm baseline diameter vs. 12.96 ± 0.8 μm after aCSF ejection, *n* = 6 experiments, 3 mice, *P* = 0.97, paired Student’s *t*-test).

**FIGURE 5:**
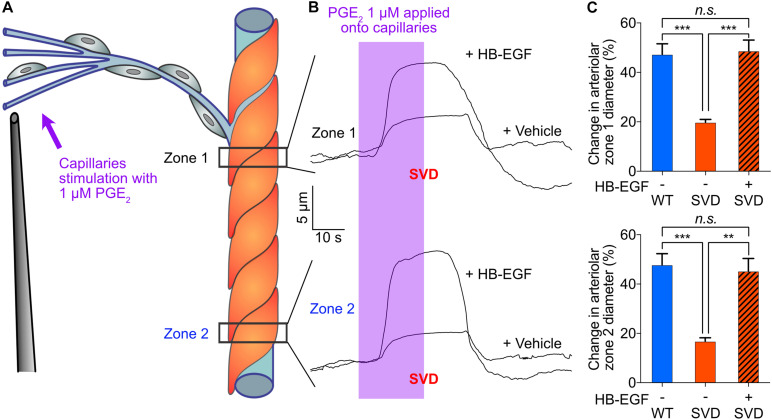
In the SVD mouse model, blunted upstream arteriolar dilation in response to capillary stimulation with PGE_2_ is completely restored by bath application of 30 ng/ml HB-EGF. **(A)** Cartoon showing Zone 1 and Zone 2 positions for arteriolar diameter recording during capillaries stimulation with 1 μM PGE2. **(B)** Representative traces of arteriolar diameter showing the effect of pressure ejection of PGE_2_ 1 μM onto capillaries in absence or presence of 30 ng/ml HB-EGF in a preparation from SVD mouse model. **(C)** Summary data from 5 to 6 mice (*n.s.*, not significant; **P < 0.01, ***P < 0.001, one-way ANOVA Dunnett’s test).

Finally, we investigated the effect of SVD on PGE_2_-initiated capillary-to-arteriole signaling. *Ex vivo* stimulation of capillaries from SVD mice with PGE_2_ induced an upstream arteriolar dilation that was 58% smaller at Zone 1 and 64.8% smaller at Zone 2 compared with that in *TgNotch3*^*WT*^ control (WT) mice, revealing attenuated PGE_2_-mediated signaling (Figure 5). The difference between zone 1 and 2 dilations were not significant. We previously reported that activation of the epidermal growth factor receptor (EGFR) by its ligand heparin-binding EGF-like growth factor (HB-EGF) ameliorates the cerebral vascular deficits of the SVD mouse—including neurovascular coupling and functional hyperemia deficits (Capone et al., 2016; Dabertrand et al., 2021). We then directly tested the effect of HB-EGF on PGE_2_-induced upstream vasodilation in *ex vivo* preparations from SVD mice. Bath-applied 30 ng/mL HB-EGF dramatically enhanced the upstream arteriolar dilation induced by capillary stimulation with PGE_2_, increasing the average dilation from 19.8% ± 1% to 48.7% ± 4.3% at Zone 1, and from 16.8% ± 1.4% to 45.3% ± 5.1% at Zone 2 (Figure 5 and Supplementary Movie 3). HB-EGF completely restored PGE_2_-induced upstream dilation, abolishing the differences measured between WT and SVD animals.

## Discussion

**FIGURE 6:**
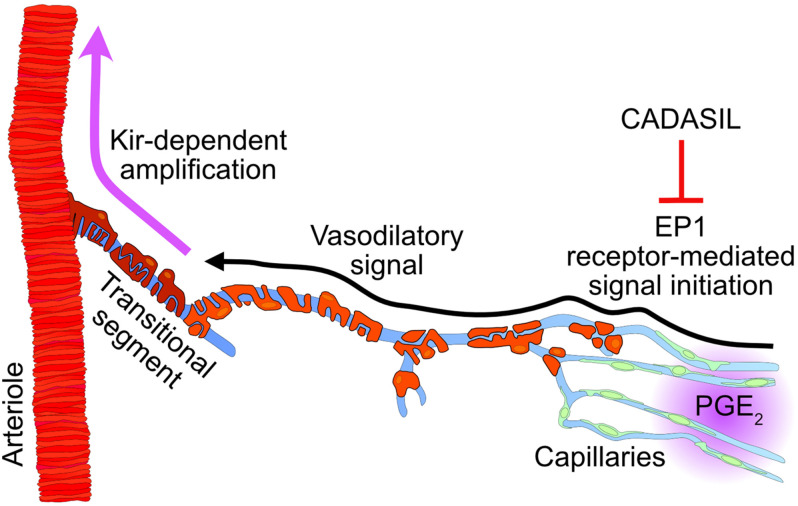
Proposed mechanism involving PGE_2_ during functional hyperemia and its inhibition in cerebral small vessel disease (CADASIL). Intracellular increases in Ca^2+^ caused by phospholipase C activation during EP1 signaling triggers a propagating vasodilatory signal that is amplified by Kir channel in the transitional segment region. CADASIL, which lowers the pool of phospholipase C substrate PIP_2_, would limit the synthesis of the second messenger IP_3_, resulting in a reduced PGE_2_-evoked upstream dilation during functional hyperemia.

Our progress in understanding functional hyperemia in health and disease has been hampered by large gaps in our comprehension of the mechanism underlying this basic physiological response—and persistent controversies surrounding it (Kaplan et al., 2020). Our recent work identified Kir2.1 channels in cECs as the molecular cornerstone initiating and propagating a retrograde hyperpolarizing vasodilatory signal from capillaries to arterioles (Longden et al., 2017; Harraz et al., 2018a; Moshkforoush et al., 2020; Dabertrand et al., 2021). The present study extends this capillary-based paradigm, providing support for a new signaling modality that posits a central role for the G_*q*_PCR EP1 receptor in mediating PGE_2_-induced vasodilatory signal that propagates upstream to cause dilation of feeding parenchymal arterioles (Figure 6). Importantly, these findings hold the promise of resolving controversies surrounding how PGE_2_, a widely proposed mediator of neurovascular coupling (Zonta et al., 2002; Takano et al., 2005; Gordon et al., 2008; Attwell et al., 2010; Watkins et al., 2014), can promote functional hyperemia despite evidence that it directly constricts arterioles (Dabertrand et al., 2013).

Our observations raise the immediate question of which effect of PGE_2_, capillary-mediated vasodilation or direct arteriolar constriction, predominates during neurovascular coupling. We calculated an EC_50_ of 145 nM for the PGE_2_-induced constriction of the parenchymal arteriole, while the vasodilation induced by capillary stimulation displayed an EC_50_ of 70 nM. In our experimental conditions, a simultaneous stimulation of capillaries and the arteriole with 500 nM PGE_2_ led to a constriction, suggesting that the arteriolar effect prevails when both vascular segments are exposed to PGE_2_. A possible explanation for these observations is that the microvascular response to PGE_2_ has a kinetic component in which the fast constriction predominates over the slower capillary-mediated response. However, this stimulation via the bath perfusion likely does not reflect *in vivo* conditions. Accordingly, we tested the effect of increasing our ejection duration *in vivo* such that PGE_2_ not only stimulated the targeted capillaries but also spread upstream to the arteriole. Here too this maneuver produced arteriolar constriction, again suggesting that this response will predominate when the arteriole is exposed directly to PGE_2_. Since the dense capillary network within the brain lies in close proximity to all neurons (Nishimura et al., 2007; Blinder et al., 2013), it is expected that cECs are the primary sensors of neuronal activity and any neurally derived PGE_2_. Therefore, the vasodilatory effect would be expected to predominate during neurovascular coupling in physiological conditions and our data suggest that this occurs through local exposure of the capillaries to PGE_2_. However, a variety of brain conditions, including ischemia and neurodegeneration (Minghetti, 2004), are known to up-regulate COX-2 expression in excitatory neurons which results in an EP1-dependent neurotoxicity of PGE_2_ (Kawano et al., 2006). In this pathological situation, higher PGE_2_ concentrations could lead to arteriolar constriction and then contribute to PGE_2_ neurotoxicity by limiting local blood supply.

Neuronal activation leads to rapid increases in blood flow within, and on the surface, of the brain. Hillman and colleagues elegantly provided evidence for involvement of the endothelium in stimulus-evoked, conducted vasodilation from the brain parenchyma to arterioles and pial arteries *in vivo* (Chen et al., 2014). Our previous study on neurovascular coupling demonstrated how capillary endothelium is capable of transmitting an electrical signal to cause upstream vasodilation in support of functional hyperemia (Longden et al., 2017). We showed that activation of the Kir2.1 channel in cECs by extracellular K^+^ ions propagates a regenerative hyperpolarization from cell-to-cell up to the feeding arteriole to cause vasodilation (Longden et al., 2017; Harraz et al., 2018a; MacMillan and Evans, 2018). Interestingly, recent work from Thakore et al. (2021) showed that 4-hydroxynonenal (4-HNE), an endogenous product of lipid peroxidation, activates transient receptor potential ankyrin 1 (TRPA1) channel in cECs to cause upstream arteriolar dilation during functional hyperemia. These recent findings introduce the concept that a slowly propagating short-range Ca^2+^ signal is initiated in the capillary endothelium and converted into the fast-propagating hyperpolarization that causes dilation of upstream arterioles. The conversion is proposed to occur in the transitional region between the capillaries and the arteriole (Ratelade et al., 2020) by activation of the small- and intermediate-conductance Ca^2+^-activated K^+^ channels (SK and IK, respectively) and amplification of the hyperpolarization by Kir channel (Thakore et al., 2021). The transitional region refers to the first segment sprouting out of the arteriole, visible on Figure 1 micrographs, while local stimulations are applied onto the 3rd and 4th order capillary branches after the transitional region. A vast body of literature reports (de Wit et al., 1999; Domeier and Segal, 2007; Bagher and Segal, 2011) supports the concept that a propagating Ca^2+^ signal is capable of acting through IK/SK channels, which are not present in cECs (Longden et al., 2017) but are expressed by transitional and arteriolar ECs (Hannah et al., 2011; Thakore et al., 2021). The generated hyperpolarizing signal is further amplified via Kir channel activation (Sonkusare et al., 2016) and conveyed through myoendothelial junctions to adjacent SMCs. In the dilation induced by capillary stimulation with PGE_2_, inhibition of the Kir2.1 channel with Ba^2+^ had a profound effect, particularly measurable on the propagation of the dilation, which is consistent with the biphasic propagative model proposed by Thakore et al. (2021). Interestingly, inhibition of TRPA1 channels did not prevent PGE_2_ from causing upstream dilation, suggesting a different initiation mechanism, likely involving G_*q*_ protein activation and inositol trisphosphate (IP_3_)-mediated Ca^2+^ release, as opposed to direct Ca^2+^ entry across the plasma membrane. Our previous work also highlighted the role of G_*q*_PCR activation in breaking down phosphatidylinositol 4,5-bisphosphate (PIP_2_), resulting in decreased Kir2.1 channel activity and increased open probability of the Ca^2+^/Na^+^-permeable TRPV4 channel (Harraz et al., 2018a,b, 2020). However, blocking TRPV4 channels had no effect on the dilation induced by PGE_2_ either. Given these data, we propose that EP1-initiated IP_3_-dependent Ca^2+^ signals arriving in the transitional segment activate endothelial IK/SK channels, and the ensuing membrane potential hyperpolarization activates Kir channels, converting the incoming Ca^2+^ signal into a Kir-dependent hyperpolarizing signal. The characterization of such a Ca^2+^ signal will require more extensive investigation, but the vasodilation induced by capillary stimulation with PGE_2_ is clearly central to the postulated role of this molecule as a neurovascular coupling agent.

Cerebral SVDs have emerged as a central link between two major co-morbidities. They account for more than 30% of strokes worldwide and at least 40% of dementia cases (Pantoni, 2010; Iadecola, 2013). CADASIL is caused by dominant mutations in the NOTCH3 receptor, expressed by SMCs and pericytes, that stereotypically lead to the extracellular deposition of the NOTCH3 ectodomain (NOTCH3^*ECD*^), which recruits and aggregates other proteins on vessels, ultimately forming deposits termed granular osmiophilic material (GOM) (Joutel et al., 2000, 2016; Chabriat et al., 2009). One of these proteins is the tissue inhibitor of metalloproteinases 3 (TIMP3), which directly complexes with NOTCH^*ECD*^ and abnormally accumulates in the extra cellular matrix of brain vessels in patients and mice with CADASIL (Monet-Leprêtre et al., 2013). A deficit in CBF hemodynamics, including functional hyperemia, is an early disease manifestation in patients (Chabriat et al., 2000; Pfefferkorn et al., 2001; Liem et al., 2009; Huneau et al., 2018) and a prominent feature of the well-established *TgNotch3^*R169C*^* CADASIL mouse model used in the present study (Joutel et al., 2010; Capone et al., 2016; Dabertrand et al., 2021). Our recent work indicates that TIMP3 effects on cerebrovascular reactivity are attributable to inhibition of ADAM17 and subsequent suppression of EGFR signaling by inhibition of ectodomain shedding of its ligand HB-EGF (Dabertrand et al., 2015, 2021; Capone et al., 2016).

Consistent with this model, we previously found that EGFR activation with exogenous soluble HB-EGF restores cerebral arterial tone and functional hyperemia (Dabertrand et al., 2015, 2021; Capone et al., 2016). Here, we found that PGE_2_-induced dilation was impaired in the CADASIL mouse model and fully restored by HB-EGF.

We previously identified two downstream consequences of the suppressed TIMP3-ADAM17-EGFR signaling module: (i) the upregulation of voltage gated K^+^ (K_*V*_1.5) channels in the arteriolar SMCs (Dabertrand et al., 2015; Capone et al., 2016); and (ii) the partial inhibition of Kir2.1 channels in cECs, but not in arteriolar ECs and SMCs (Dabertrand et al., 2021). Here, we report a third consequence: the disruption of PGE_2_-induced capillary-to-arteriole signaling, reinforcing the concept that extracellular matrix alterations have profound impacts on cerebrovascular dynamics in SVDs. Using computational modeling, we previously investigated the impact of K_*V*_ channel upregulation on membrane potential dynamics in the context of concurrent activation of myocyte Kir channels (Koide et al., 2018). Interestingly, while these analyses showed that a higher K_*V*_ channel current density would reduce the membrane potential range over which Kir channels can be activated to cause and propagate dilation, the more hyperpolarized resting membrane potential (9 mV) actually facilitates Kir channel activation. Thus, arterioles from the CADASIL mouse model would still hyperpolarize and dilate in response to Kir channel opening, as observed experimentally (Dabertrand et al., 2015; Koide et al., 2018), but at the cost of a smaller vasodilatory reserve. At the capillary level, we previously described, and modeled, how a 50% reduction in cECs Kir2.1 current is sufficient to completely abolish the capillary-to-arteriole electrical signaling in response to 10 mM K^+^ (Harraz et al., 2018a; Moshkforoush et al., 2020), and then strongly reduces functional hyperemia in the CADASIL mouse model (Dabertrand et al., 2021). We attributed this endothelial dysfunction to a reduced cEC metabolism caused by inhibition of the EGFR pathway (Dabertrand et al., 2021). The resulting lower ATP/ADP ratio in CADASIL compared to WT cECs decreases the synthesis of PIP_2_ and its availability to act as an essential cofactor for Kir2.1 channel—hence reducing the channel activity (Huang et al., 1998; Hansen et al., 2011; Harraz et al., 2018a, 2020). Of particular note, PIP_2_ is also a substrate for phospholipase C during EP1 signaling, and a reduced pool of PIP_2_ would certainly limit the synthesis of the second messenger IP_3_, which mobilizes Ca^2+^ from endoplasmic reticulum stores through its action on its cognate receptor. The observation that HB-EGF restores both Kir2.1- and PGE_2_-initiated signaling is consistent with this concept. The impact of CADASIL on neurovascular coupling is thus multifaceted, involving disruption of two different propagating signals sharing connections to the EGFR pathway in cECs, and Kir channel activation in the transitional and arteriolar segments.

We have made major progress in establishing potential contributing mechanisms to cerebral hemodynamics impairment observed at an early stage of CADASIL, a Mendelian paradigm of SVDs (Chabriat et al., 2009) and the most common hereditary cause of stroke (Pantoni, 2010) and dementia (Schmidt et al., 2012). Here we demonstrate that functional hyperemia deficits in this SVD involve a PGE_2_-initiated capillary-to-arteriole signal that is normally regulated by the TIMP3-ADAM17-EGFR signaling module. Furthermore, the evidence that PGE_2_ induces arteriolar dilation via capillary stimulation has the potential to reconcile disparate findings in neurovascular studies. Our mechanistic studies thus lay the groundwork for novel targeted strategies for treating CADASIL and other cerebrovascular diseases.

## Data Availability Statement

The original contributions presented in the study are included in the article/Supplementary Material, further inquiries can be directed to the corresponding author.

## Ethics Statement

The animal study was reviewed and approved by the Institutional Animal Care and Use Committee (IACUC) of the University of Colorado, Anschutz Medical Campus.

## Author Contributions

AR, JF, and FD performed *ex vivo* experiments, data collection, and analysis. TL and NW performed *in vivo* experiments, data collection, and analysis. AJ contributed to the study design. FD designed and directed the research and wrote the manuscript. All authors edited the manuscript and approved its submission.

## Conflict of Interest

The authors declare that the research was conducted in the absence of any commercial or financial relationships that could be construed as a potential conflict of interest. The reviewer GW declared a shared affiliation, with no collaboration, with one of the authors AJ to the handling editor at the time of the review.

## Publisher’s Note

All claims expressed in this article are solely those of the authors and do not necessarily represent those of their affiliated organizations, or those of the publisher, the editors and the reviewers. Any product that may be evaluated in this article, or claim that may be made by its manufacturer, is not guaranteed or endorsed by the publisher.

## Supplementary Material

Supplementary Movie 1 Capillary stimulation with PGE2 1 μM evokes upstream arterial dilation in *ex vivo* CaPA preparation.

Supplementary Movie 2 Capillary stimulation with PGE_2_ causes upstream dilation of the arteriole and transitional segment *in vivo*.

Supplementary Movie 3 Exogenous application of 30 ng/ml HB-EGF fully restores upstream arterial dilation in response to capillary stimulation with 1 μM PGE_2_ in CaPA preparation from a SVD mouse.
